# The Utility of Addenbrooke’s Cognitive Examination III (ACE-III) in Differentiating Neurodegenerative Disorders with Psychotic Symptoms: A Narrative Review

**DOI:** 10.3390/healthcare14101313

**Published:** 2026-05-12

**Authors:** Anna Barczak

**Affiliations:** Rare and Civilization Diseases Research Platform, Mossakowski Medical Research Institute, Polish Academy of Science, 5 Pawiński Str., 02-106 Warsaw, Poland; abarczak@imdik.pan.pl; Tel.: +48-22-6086482

**Keywords:** Addenbrooke’s Cognitive Examination III (ACE-III), cognitive screening, neurodegenerative disorders, psychosis, Alzheimer’s disease, dementia with Lewy bodies, Parkinson’s disease dementia, Frontotemporal dementia, differential diagnosis

## Abstract

Psychotic symptoms, including delusions and hallucinations, frequently complicate the course of Alzheimer’s disease (AD), dementia with Lewy bodies (DLB), Parkinson’s disease dementia (PDD), and frontotemporal dementia (FTD). Their presence accelerates decline, worsens outcomes, and complicates management. Cognitive screening in such patients is essential yet challenging due to attentional fluctuation, impaired insight, and diagnostic overlap. Addenbrooke’s Cognitive Examination III (ACE-III) is a multidomain tool with higher sensitivity than the MMSE. Evidence indicates that ACE-III captures disorder-specific cognitive-psychotic profiles: memory impairment in AD with delusions of theft, visuospatial and attentional deficits in DLB with hallucinations, or executive dysfunction in FTD with paranoid ideation. Mini-ACE (M-ACE), a shorter derivative, is useful in acute psychiatric or advanced dementia settings. This review synthesizes evidence on ACE-III and M-ACE in psychosis-related neurodegeneration, highlights their role in differentiating from primary psychiatric psychoses, and identifies knowledge gaps, particularly in atypical AD variants, mixed dementia, and autosomal dominant AD. ACE-III emerges as a practical and informative tool, but psychosis-specific normative data and longitudinal studies are needed.

## 1. Introduction

### The Diagnostic Challenge of Psychosis in Older Adults

Psychotic symptoms emerging in later life constitute a major diagnostic challenge, as hallucinations and delusions may reflect either a primary psychiatric disorder or an early manifestation of an underlying neurodegenerative process [1]. In older adults, the phenomenology of psychosis often overlaps across diagnostic categories, and reliance on psychiatric symptom profiles alone is frequently insufficient for accurate etiological diagnosis. Misclassification may result in inappropriate treatment, delayed recognition of dementia, and exposure to potentially harmful interventions [2].

Not all neurodegenerative illnesses begin late in life. Some, such as frontotemporal dementia (FTD), can start in mid-adulthood or early old age. These cases often precede the common age range for Alzheimer’s disease (AD) [3]. Early on, psychotic and behavioral symptoms may dominate, making misdiagnosis as a primary psychiatric disorder more likely. Accurate diagnosis is especially crucial in younger patients with psychosis, as delays in recognizing neurodegeneration can worsen management, prognosis, and caregiver burden [2,4].

Psychosis, manifested by delusions and hallucinations, constitutes a core component of the behavioral and psychological symptoms of dementia (BPSDs). In some patients, psychotic features may precede overt cognitive impairment or represent a central clinical manifestation of neurodegenerative disease [1]. More commonly, however, psychotic symptoms increase in frequency and severity as neurodegeneration progresses [5]. Psychosis in dementia is highly prevalent, affecting up to 63% of patients [6], and is associated with increased distress, accelerated cognitive and functional decline, higher rates of institutionalization, and increased mortality [7].

Early etiological diagnosis of neurodegenerative disease is therefore essential not only for prognostic accuracy but also for anticipating neuropsychiatric complications and guiding management. Although hallucinations and delusions occur across multiple neurodegenerative disorders, their timing, phenomenology, and clinical implications vary depending on the underlying pathology [8,9]. Diagnostic frameworks, including the International Psychogeriatric Association criteria and the Alzheimer’s Association (ISTAART) research criteria, emphasize the importance of identifying psychosis independently of cognitive stage [10,11].

In this complex diagnostic landscape, cognitive assessment plays a central role. Brief screening tools such as the Mini-Mental State Examination (MMSE) [12] and the Montreal Cognitive Assessment (MoCA) [13] may not adequately capture profile-based patterns necessary for differentiating dementia syndromes and distinguishing neurodegenerative from primary psychiatric conditions [14]. Importantly, tools that assess domain-specific cognitive profiles rather than global performance are particularly valuable in this context. Cognitive assessment should be interpreted within a broader diagnostic framework that also includes functional measures, such as the Functional Activities Questionnaire (FAQ) [15], neuropsychiatric tools such as the Neuropsychiatric Inventory Questionnaire (NPI-Q) [16], and global staging instruments such as the Clinical Dementia Rating (CDR) [17]. These complementary measures provide essential context for interpreting cognitive findings and support more accurate etiological diagnosis.

Multidomain instruments, such as Addenbrooke’s Cognitive Examination III (ACE-III) [18], provide a structured, time-efficient evaluation of attention, memory, language, fluency, and visuospatial abilities. By identifying characteristic cognitive patterns, ACE-III may improve diagnostic accuracy in patients presenting with psychotic symptoms, particularly when psychiatric features obscure early cognitive decline [19]. Within this broader diagnostic framework, ACE-III offers detailed cognitive profiling that complements functional and neuropsychiatric assessment.

Despite growing interest in cognitive profiling in neurodegenerative disorders, there remains a lack of integrative synthesis focusing specifically on the use of multidomain cognitive tools in patients presenting with psychosis [20]. In particular, the relationship between characteristic cognitive patterns and neuropsychiatric symptomatology remains underexplored [21]. 

Accordingly, this review examines the clinical utility of ACE-III in the differential diagnosis of neurodegenerative disorders and discusses its role in distinguishing dementia-related psychosis from primary psychiatric conditions across the adult lifespan.

From a neurobiological perspective, cognitive profiles identified with ACE-III can be interpreted within the framework of large-scale brain network dysfunction. AD predominantly affects medial temporal and hippocampal networks, leading to episodic memory impairment. Dementia with Lewy bodies (DLB) involves posterior cortical and attentional networks, resulting in visuospatial and attentional deficits. Parkinson’s disease dementia (PDD) is associated with frontostriatal circuit dysfunction, reflected in executive impairment, while FTD primarily affects frontal and anterior temporal networks, leading to executive and language deficits [22,23]. This network-based framework provides a mechanistic basis for understanding why multidomain cognitive patterns observed in ACE-III support differential diagnosis across neurodegenerative disorders and align with contemporary models of network-based neurodegeneration.

## 2. Methods

This narrative review was based on a structured literature search in PubMed, Scopus, and Web of Science. The search used combinations of these terms: “ACE-III”, “Addenbrooke’s Cognitive Examination”, “Mini-ACE”, “cognitive screening”, “psychosis”, “neurodegenerative disorders”, “Alzheimer’s disease”, “Lewy body dementia”, “Parkinson’s disease dementia”, and “frontotemporal dementia”. 

We considered studies published in English from 2000 to 2025. Eligible studies included original research articles, systematic reviews, and meta-analyses that addressed cognitive assessment with the ACE-III or Mini-ACE (M-ACE) in neurodegenerative or psychiatric populations. It should be noted that the ACE-III has limitations, including potential ceiling or floor effects and reduced sensitivity in certain cultural or educational contexts. Selection was based on relevance to clinical application, cognitive profiling, and differential diagnosis. 

Since this is a narrative review, formal systematic selection procedures were not applied. Still, efforts were made to represent key studies fairly and to include both primary research and review articles. Some non-English sources were also included if they offered unique methodological contributions. For example, Sitek et al. (2017) [24], published in Polish, was included because it provides a percentage-based analysis of ACE-III performance that is rarely reported in English sources.

## 3. Addenbrooke’s Cognitive Examination III as a Multidomain Diagnostic Tool

### 3.1. Addenbrooke’s Cognitive Examination III—ACE-III

The ACE-III is a brief cognitive screening tool suitable for clinical practice. Administration typically takes 20 min and yields a total score from 0 to 100, with higher scores indicating better cognitive function. The ACE-III evaluates domains such as attention, memory, verbal fluency, language, and visuospatial skills, helping clinicians identify specific cognitive impairments. The Attention domain (18/100) measures orientation, immediate repetition, and serial subtraction. The Memory domain (26/100) covers delayed recall, learning, recall of a fictional name and address, recognition memory, and factual recall. Verbal Fluency (14/100) tests word generation (by initial letter and animal category). The Language domain (26/100) includes comprehension of multi-step commands, sentence writing, repetition, naming, semantic comprehension, and reading irregular words. Visuospatial skills (16/100) are evaluated via figure copying, clock drawing, dot counting, and recognition of fragmented letters [18,25].

ACE-III domain scores closely match established neuropsychological tests, supporting its use as a multidomain tool [25,26,27]. ACE-III scores can also predict results on broader neuropsychological test batteries [28].

ACE-III determines the severity of cognitive impairment [25,26,28] and outperforms shorter screening instruments in identifying clinically significant cognitive deficits [25,29,30,31]. It also enables recognition of cognitive profiles characteristic of various neurocognitive disorders [28,32,33,34,35,36], while facilitating monitoring of meaningful cognitive changes throughout disease progression [37].

The diagnostic utility of ACE-III extends beyond the total score. Specifically, qualitative analysis of domain-level performance can reveal cognitive dissociations not detected by global cut-off values [24,36]. This profile-based approach, therefore, helps differentiate dementia subtypes and identify neurodegenerative diseases. Moreover, it is especially valuable for patients whose initial presentation involves neuropsychiatric, rather than cognitive, symptoms.

ACE-III is used to assess cognition in patients with primary psychotic disorders [19,38,39,40] and is incorporated into research protocols investigating cognitive functioning in psychosis [4,41]. In these populations, ACE-III helps characterize cognitive performance and supports differentiation between neurodegenerative and primary psychiatric etiologies when interpreted within broader clinical and longitudinal frameworks.

In clinical settings, ACE-III is more than a quantitative screening tool; it also serves as a structured cognitive profiling instrument. Furthermore, the distribution of scores across domains often provides more diagnostically relevant information than the total score [24]. Domain-specific dissociations may indicate underlying neurodegenerative processes, and these results should be interpreted within the broader clinical context [36].

ACE-III enables partial comparison of cognitive domains using percent scores, which may facilitate differentiation between dementia syndromes [24]. This approach is particularly valuable in patients with psychotic symptoms, where global cognitive function may appear intact despite significant cognitive profile abnormalities.

### 3.2. Mini-Addenbrooke’s Cognitive Examination (M-ACE)

The M-ACE is a brief screening tool derived from ACE-III, designed to rapidly assess global cognitive functioning while remaining sensitive to clinically relevant impairment patterns. The tool consists of four attention questions, animal fluency, address learning and recall, and the clock drawing task [42]. Administration takes about 5 to 10 min and produces a score from 0 to 30. These features offer practical advantages for busy clinical settings, making M-ACE an effective first-line instrument in the diagnostic pathway for patients with cognitive or neuropsychiatric symptoms [43,44].

M-ACE offers greater sensitivity and specificity for dementia than the MMSE, making it especially valuable for clinicians detecting early cognitive impairment [29,42,43,44,45,46]. While it lacks the full multidomain coverage of ACE-III, M-ACE assesses memory, fluency, visuospatial abilities, and attention, supporting initial identification of atypical cognitive profiles in clinical practice.

For differential diagnosis, M-ACE is best regarded as a triage tool within a stepped diagnostic pathway. Abnormal results should prompt a comprehensive cognitive assessment, such as ACE-III or a full neuropsychological evaluation. In contrast, preserved M-ACE scores—particularly in patients presenting with psychotic symptoms—may support consideration of a primary psychiatric disorder; however, longitudinal monitoring remains essential, as early neurodegenerative disease can present with subtle or fluctuating deficits [29].

Although M-ACE is shorter and less detailed than ACE-III, its main clinical value lies in the rapid identification of cognitive impairment and guidance of subsequent diagnostic steps. Rather than differentiating dementia subtypes or establishing etiology, M-ACE helps clinicians determine which patients require immediate, extensive evaluation and which can initially be monitored within a structured follow-up pathway. In this way, M-ACE complements ACE-III by improving workflow efficiency while maintaining diagnostic vigilance.

## 4. Cognitive Profiles of Major Dementia Syndromes Assessed with ACE-III

The ACE-III enables multidomain cognitive profiling, especially useful for differentiating dementia syndromes, as distinct patterns of domain vulnerability reflect underlying neuropathological processes. Interpretation of ACE-III results should therefore emphasize a qualitative, domain-level examination rather than reliance on global cut-off scores alone.

### 4.1. ACE-III in Alzheimer’s Disease

The ACE-III profile of AD with memory-dominant impairment is generally marked by early and prominent impairment in episodic memory, particularly delayed recall, recognition, and learning tasks, with subsequent involvement of language and orientation. Verbal fluency is often reduced, with semantic fluency more affected than phonemic fluency in more advanced stages, reflecting degradation of semantic memory networks. Except for working memory and constructional tasks, visuospatial abilities, and attention are relatively preserved in early stages but decline as the disease progresses. This memory-led cognitive profile distinguishes AD from other dementia syndromes and is usually evident on ACE-III even when the total score remains near diagnostic thresholds. As disease severity increases, deficits become more global, reducing the specificity of the profile [18,25,26,35].

#### Cognitive Profiles of Atypical Alzheimer’s Disease Variants Assessed with ACE-III

Atypical variants of AD present with non-amnestic cognitive profiles reflecting selective vulnerability of posterior, language, frontal, or motor-related cortical networks. In these syndromes, multidomain assessment with the ACE-III is particularly informative, as reliance on global cognitive scores or memory-dominant screening tools may obscure diagnostically relevant dissociations. Qualitative, domain-level interpretation of ACE-III performance is therefore essential for accurate etiological recognition [24,27,28]. Table 1 presents cognitive and psychotic features across neurodegenerative disorders relevant to ACE-III interpretation.

### 4.2. ACE-III in Dementia with Lewy Bodies (DLB)

DLB demonstrates a characteristic ACE-III profile defined by disproportionate impairment in visuospatial abilities and attention, often accompanied by cognitive fluctuations. Tasks involving figure copying, clock drawing, dot counting, and visual perception are frequently impaired early, while episodic memory may be relatively preserved compared with AD at similar stages. Attention scores may vary substantially across testing sessions, reflecting intrinsic cognitive fluctuations. Verbal fluency and executive functions are also commonly affected [24,32,37]. This visuospatial–attention-led profile matches closely the high prevalence of visual hallucinations and perceptual disturbances observed in DLB.

### 4.3. Parkinson’s Disease Dementia (PDD) Profile in ACE-III

In PDD, ACE-III typically reveals prominent executive and attentional dysfunction, with secondary impairment in visuospatial abilities. Verbal fluency—both phonemic and semantic—is often reduced, reflecting involvement of the frontostriatal circuit. Episodic memory deficits are usually milder than in AD and may be partly related to retrieval inefficiency rather than encoding failure [24,32,37]. Compared with DLB, visuospatial deficits in PDD are often less pronounced in early stages, while the cognitive profile is more strongly weighted toward executive dysfunction. This pattern supports differentiation between PDD and AD, particularly when interpreted in the context of motor symptoms and disease chronology.

Longitudinal assessment may further enhance interpretation. A shift from a predominantly executive–attentional profile toward more generalized deficits, including memory and more pronounced visuospatial impairment, may suggest the presence of mixed or additional pathology. However, such observations require confirmation with biomarkers, neuroimaging, and longitudinal clinical evaluation, as ACE-III alone is insufficient for etiological diagnosis [36].

### 4.4. ACE-III in Frontotemporal Dementia (FTD)

Cognitive profiles on ACE-III in FTD vary by clinical subtype but are unified by disproportionate executive and fluency impairment with relative sparing of episodic memory in early stages.

Behavioral variant FTD (bvFTD): marked reductions in verbal fluency and executive control, often with near-normal memory and visuospatial performance early on. Total ACE-III scores may be misleadingly preserved.

Semantic variant PPA: profound impairment in naming and semantic comprehension within the language domain, with better letter fluency compared to the semantic one, repetition, and visuospatial abilities.

Non-fluent/agrammatic PPA: deficits in speech production, repetition, and syntactic comprehension, with relatively preserved semantic knowledge (naming) and memory.

Qualitative domain dissociations on ACE-III are therefore critical for recognizing FTD and distinguishing it from AD and primary psychiatric disorders [4,24,35].

Beyond behavioral variant FTD and the classical language-led primary progressive aphasia (PPA) syndromes, several additional FTD-related clinical phenotypes may be encountered in specialist practice. Although these variants are less common, they are diagnostically important, as their presentations may overlap with atypical AD, movement disorders, or primary psychiatric conditions. Multidomain cognitive assessment with the ACE-III, when interpreted qualitatively, can provide useful support for differential diagnosis by highlighting selective domain vulnerabilities. Across less common FTD variants, ACE-III is most effective for diagnosis when used as a profile-based tool rather than a simple screening measure. Preservation of episodic memory in the presence of executive, semantic, or fluency-led deficits should prompt consideration of FTD-spectrum disorders, even when total scores fall within normal limits. Integration of ACE-III findings with behavioral history, neuroimaging, genetic testing, and longitudinal observation remains essential for accurate etiological diagnosis [18,24,35]. 

### 4.5. Vascular Dementia (VaD) Profile in ACE-III

VaD is characterized by heterogeneous, “patchy” cognitive profiles on the ACE-III, reflecting the distribution and burden of cerebrovascular pathology. Executive dysfunction, attentional deficits, and psychomotor slowing are typically prominent, while memory impairment is variable and often less severe than in AD. Performance across domains may be uneven, and a stepwise decline may be observed over time. This non-uniform profile differentiates VaD from the more stereotyped patterns seen in AD and DLB and highlights the importance of integrating ACE-III findings with neuroimaging evidence of vascular disease [24,36,47].

Across dementia syndromes, ACE-III supports etiological differentiation by uncovering specific cognitive signatures: memory-led impairment in AD, visuospatial-attention deficits in DLB, executive dysfunction in PDD and FTD, and heterogeneous executive-attentional profiles in VaD [48]. Qualitative, profile-based interpretation of ACE-III domains is therefore crucial for accurate clinical reasoning, particularly in patients presenting with neuropsychiatric symptoms or atypical disease onset.

The major neurodegenerative syndromes associated with psychotic symptoms differ substantially in cognitive profile, phenomenology of psychosis, and behavioral presentation. A comparative overview of the principal clinical characteristics relevant to ACE-III interpretation is presented in Table 1.

## 5. Psychosis in Neurodegenerative Disorders

Identification of distinct psychotic features across neurodegenerative disorders allows for more accurate differential diagnosis and facilitates disorder-specific management, thereby improving patient outcomes.

In DLB and PDD, early psychotic symptoms—especially visual hallucinations—are common. In AD, psychosis mainly emerges in moderate to advanced stages, signaling accelerated decline and worse prognosis. Early behavioral disturbances and delusional ideation in bvFTD may lead to psychiatric misdiagnosis, highlighting the need for careful evaluation. Psychotic symptoms in MCI indicate increased dementia risk, supporting close monitoring and early intervention.

### 5.1. Psychosis in Alzheimer’s Disease

In AD, delusions are the most common psychotic symptoms, while hallucinations—typically visual—occur less often and usually emerge later [9]. These symptoms appear mainly in moderate to advanced stages and are associated with faster cognitive decline, poorer outcomes, and increased mortality [8]. The overall prevalence is 30–50%, though estimates vary by stage and methodology [7]. Delusions often involve persecution or misidentification, whereas hallucinations (5–15%) increase with disease progression [6].

Psychotic symptoms appear more frequently in late-onset AD than in early-onset forms [49]. In genetically determined AD, particularly in PSEN mutation carriers, psychosis may occur early and mimic primary psychiatric disorders [50,51].

Among atypical AD variants, PCA stands out for early and recurrent visual hallucinations, closely resembling DLB, while other atypical AD presentations show less notable psychotic symptoms, which mainly appear at later stages—clarifying a key distinction between these subtypes [52,53].

### 5.2. Psychosis in Mild Cognitive Impairment

Psychotic symptoms are relatively uncommon in MCI but are clinically significant, as they are associated with an increased risk of progression to dementia [7,54]. Within the framework of Mild Behavioral Impairment (MBI), psychosis is considered a potential early marker of neurodegeneration, particularly when persistent and emerging later in life [7,55].

### 5.3. Psychosis in Dementia with Lewy Bodies

DLB is characterized by a high prevalence of psychotic symptoms, particularly early and well-formed visual hallucinations occurring in up to 80–90% of patients [56,57]. Delusions are also common (≈50–55%), typically with paranoid or misidentification themes [58]. Minor hallucinations may precede overt dementia and serve as early clinical markers [56,57,59].

Psychosis in DLB necessitates vigilant antipsychotic management because of high sensitivity and significant risks. Early onset, visual predominance, cognitive fluctuations, and REM sleep behavior disorder serve as key diagnostic indicators. Recognition of these features enables rapid and accurate differentiation, informing safe and individualized treatment [60].

### 5.4. Psychosis in Parkinson’s Disease Dementia

Psychotic symptoms occur in 50–80% of patients with PDD [7]. Visual hallucinations are most common, often beginning as minor phenomena and progressing to complex hallucinations, with insight declining over time [61]. Multimodal hallucinations are associated with greater disease severity and caregiver burden [62].

Delusions (40–55%) in PDD typically emerge later and are often persecutory or jealousy-related, signaling dementia progression and worse outcomes. High antipsychotic sensitivity necessitates careful selection and adjustment of dopaminergic therapy to avoid adverse effects [63].

### 5.5. Psychosis in Frontotemporal Dementia

Psychotic symptoms are not a core feature of FTD but are clinically significant, especially in bvFTD, where they may mimic primary psychiatric disorders and lead to misdiagnosis. Unlike the prominent hallucinations of DLB and PDD, psychosis in bvFTD generally differs in its presentation [64].

Delusions occur in approximately 20–30% of FTD patients, while hallucinations are less frequent and usually appear later, contrasting with the earlier and more prominent hallucinations seen in DLB and PDD [65]. Psychosis is more common in genetically determined FTD, especially in C9orf72 mutation carriers [66]. In bvFTD, psychosis often presents differently: suspiciousness, impaired insight, emotional blunting, and disturbed social cognition are typical, whereas hallucinations are generally not prominent [65]. Negative symptoms and formal thought disorder may help distinguish bvFTD from primary psychosis [67].

Psychosis in bvFTD complicates the diagnostic process. Accurate clinical differentiation requires integration of behavioral changes, executive dysfunction, and longitudinal cognitive profiling, rather than reliance solely on psychotic symptoms. This comprehensive approach helps prevent misdiagnosis and guides appropriate treatment.

### 5.6. Psychosis in Vascular Dementia

Psychotic symptoms in vascular dementia (VaD) are heterogeneous and often fluctuate, reflecting cerebrovascular burden. Unlike the more stereotyped patterns in neurodegenerative dementias, VaD psychosis may emerge abruptly after vascular events. Delusions (15–40%) and hallucinations (10–30%) vary by disease stage and subtype, with lesion location and frontal–subcortical disruption influencing the patterns [68,69,70,71].

Psychosis in VaD requires both cognitive evaluation and neuroimaging. Management should prioritize vascular risk control and careful selection of psychotropic medications to minimize complications [72].

Major neurodegenerative syndromes associated with psychotic symptoms differ in both the phenomenology of psychosis and their characteristic cognitive profiles and behavioral presentations. Table 1 provides a comparative overview of the principal clinical features relevant to ACE-III interpretation.

## 6. Late-Life Psychotic Features

Psychotic symptoms are rare among cognitively normal older adults. Delusions occur in 0.8% and hallucinations in 0.3%, but their presence confers the highest hazard ratio for incident dementia among neuropsychiatric symptoms (HR ≈ 3.6) [7,8,73].

Late-life psychosis is a heterogeneous condition that includes psychiatric, neurodegenerative, cerebrovascular, and medical causes. It is defined by the onset of hallucinations and/or delusions after age 60, or after 75 for very-late-onset cases. Compared to early-onset psychosis, late-life psychosis is more frequently associated with underlying brain pathology and medical comorbidity, which complicates diagnosis [74]. The phenomenology of late-life psychosis differs from younger-onset cases: older adults more often experience less systematized, predominantly persecutory or misidentification-related delusions, and visual or multimodal hallucinations, particularly when cognitive or sensory impairment is present. In contrast, younger patients typically exhibit more systematized delusions and pronounced affective distress, while insight in older adults may be partially preserved in the early stages.

Distinguishing late-life psychosis from dementia relies on symptom timing, cognitive trajectory, and treatment tolerance. A comprehensive assessment ensures an accurate diagnosis and appropriate care planning [75].

## 7. ACE-III in Differentiating Neurodegeneration-Related Psychosis from Primary Psychotic Disorders

Distinguishing dementia-related psychosis from primary psychotic disorders is a complex and clinically important challenge in neuropsychiatric practice [1]. Hallucinations and delusions are often prominent at the onset of both conditions. Early cognitive changes can be subtle, fluctuate, or be hidden by psychiatric symptoms. In these cases, symptom phenomenology alone is not enough to make an accurate etiological diagnosis [8].

Multidomain cognitive assessment with ACE-III offers a key diagnostic approach to this problem. Instead of measuring only global cognitive impairment, the ACE-III evaluates characteristic cognitive patterns. These patterns reflect neural network dysfunction and disease-specific vulnerability [18,26]. Such cognitive profiles serve as objective markers that help differentiate neurodegenerative from primary psychiatric psychoses.

In clinical practice, diagnosing psychosis in patients with suspected cognitive impairment requires a structured and multidimensional approach integrating cognitive, functional, and neuropsychiatric assessment [73]. Individual instruments such as ACE-III, Mini-ACE, FAQ, NPI-Q, and CDR provide complementary but distinct clinical information, and accurate etiological diagnosis depends on their combined interpretation rather than reliance on a single measure. To facilitate comparison of these tools and clarify their respective diagnostic roles, their principal characteristics, strengths, and limitations are summarized in Table 2.

**Figure 1:**
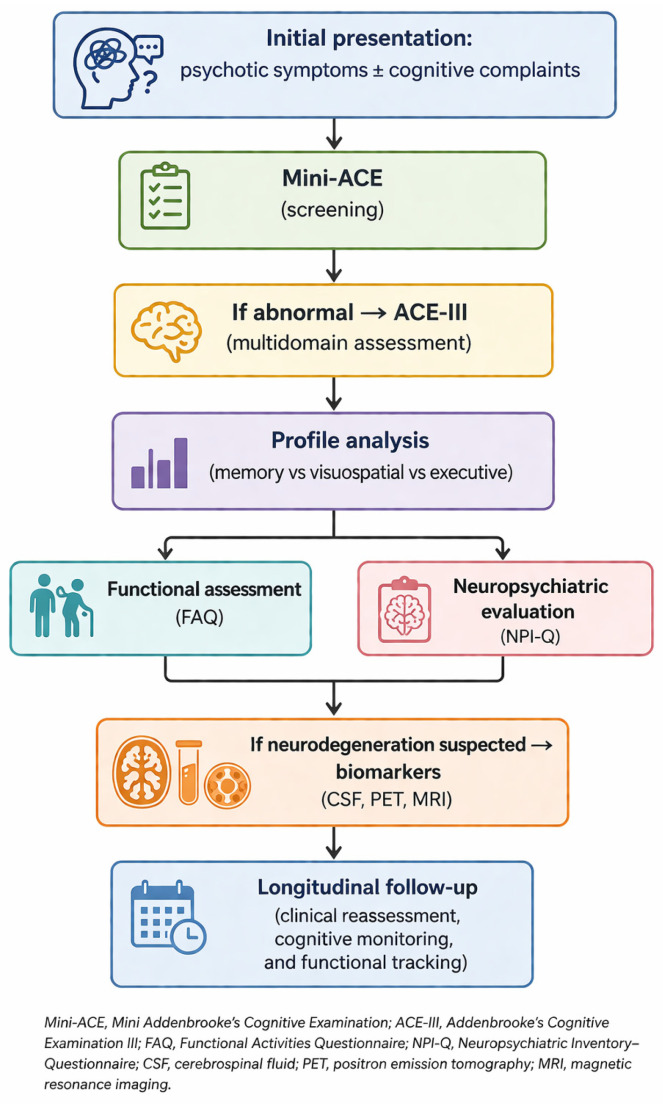
Proposed clinical assessment pathway for patients presenting with psychotic symptoms and suspected cognitive impairment. The figure was created with the assistance of generative artificial intelligence and subsequently reviewed and edited by the author.

Figure 1 presents a simplified diagnostic pathway that integrates these elements. This pathway begins with a brief cognitive screening. It then moves stepwise to multidomain evaluation, functional and behavioral assessment, and, when needed, biomarker investigation and follow-up.

### 7.1. Advantages of ACE-III in the Differential Diagnosis of Dementias with Psychotic Features

Accurate diagnosis of dementia with psychosis requires cognitive assessments that extend beyond global screening. The ACE-III offers several advantages over the MMSE and MoCA, particularly in the context of psychosis-associated dementias. 

First, ACE-III provides a comprehensive multidomain cognitive profile, assessing attention, memory, verbal fluency, language, and visuospatial abilities in a structured manner. This allows identification of syndrome-specific dissociations—such as memory-led impairment in AD, visuospatial-attentional deficits in DLB, or executive-fluency impairment in FTD—that are often obscured by total scores on the MMSE or MoCA [18,26].

Second, ACE-III is particularly well-suited to qualitative and profile-based analysis, which is essential in patients presenting with psychotic symptoms. In such cases, global cognitive severity may be mild or fluctuating, and reliance on cut-off scores alone may lead to misclassification. ACE-III domain-level and subtest-level performance enables clinicians to distinguish neurodegenerative cognitive patterns from the more inconsistent and nonspecific cognitive deficits [24] typically observed in primary psychotic disorders. This advantage is less pronounced with MMSE and MoCA, which are primarily designed as screening tools rather than instruments for etiological reasoning.

Third, ACE-III demonstrates greater diagnostic accuracy in differentiating dementia subtypes than MMSE and comparable or superior performance to MoCA, particularly when interpreted qualitatively. Quantitative studies indicate that ACE-III demonstrates higher sensitivity and specificity than MMSE in detecting mild cognitive impairment and dementia, with sensitivity often exceeding 0.85 among different cut-off values and populations. M-ACE also shows good diagnostic accuracy, although variability across studies should be considered. Studies have shown that ACE-III outperforms MMSE in detecting non-amnestic and atypical presentations of dementia [18,25]. This is clinically relevant in psychosis-associated dementias, where visuospatial, attentional, or executive dysfunction may be more informative than memory impairment alone.

The ACE-III is practical for clinical use, requiring approximately 20 min to administer. Its balance of breadth, depth, and efficiency enables it to bridge the gap between brief screening tools and comprehensive neuropsychological assessments. This feature is particularly valuable in complex cases involving psychosis.

In summary, the ACE-III is more effective than the MMSE and MoCA for diagnosing dementias with psychotic features. It facilitates multidomain, qualitative, and syndrome-oriented cognitive profiling, an approach that is essential when psychosis is the predominant clinical feature.

#### Interpretation of Reduced ACE-III Performance

A key clinical question is whether reduced ACE-III performance indicates neurodegeneration or a transient cognitive disturbance. Such disturbances may include delirium, acute psychosis, or medication effects. Several features help make this distinction.

Neurodegenerative disorders typically produce stable, internally consistent, and progressively worsening syndrome-related cognitive deficits. In contrast, transient cognitive impairment is often characterized by fluctuations, inconsistent performance across domains, and improvement following resolution of the underlying condition. Longitudinal assessment is therefore essential. Repeated ACE-III testing demonstrating progressive decline supports a neurodegenerative process, whereas improvement or variability suggests non-degenerative causes. Integration with functional assessment and clinical context remains crucial.

### 7.2. Comparison with Other Cognitive and Clinical Tools

Cognitive and neuropsychiatric assessment in patients with suspected neurodegenerative disorders requires the integration of multiple complementary instruments, each targeting different aspects of clinical presentation. MMSE is a widely used brief screening tool focused primarily on global cognitive performance, with limited sensitivity to executive and visuospatial dysfunction [12]. In contrast, ACE-III provides a multidomain cognitive profile, enabling identification of syndrome-specific patterns of impairment, which is especially useful in differentiating dementia subtypes and in patients with psychotic symptoms. The CDR scale serves a different purpose, staging dementia severity based on cognitive and functional performance [16]. It does not provide detailed cognitive domain analysis and should therefore be used alongside cognitive screening tools such as ACE-III. The FAQ [15] assesses instrumental activities of daily living and reflects functional decline, which is essential for distinguishing mild cognitive impairment from dementia, but does not capture cognitive profiles. The NPI-Q [17] evaluates behavioral and psychological symptoms, including hallucinations and delusions. While it is crucial for characterizing psychosis, it does not provide information on cognitive functioning. Taken together, ACE-III and M-ACE should be considered central cognitive tools within a broader diagnostic framework that includes MMSE (global screening), CDR (disease staging), FAQ (functional assessment), and NPI-Q (neuropsychiatric evaluation).

### 7.3. ACE-III in Differentiating Dementia from Primary Psychotic Disorders

In dementia-related psychosis, ACE-III often shows selective and consistent impairments in specific cognitive domains. These deficits form predictable patterns that match known neurodegenerative syndromes [24,37,76]. Even if total ACE-III scores are above cut-offs, the distribution of deficits can strongly suggest neurodegeneration [18].

Patients with primary psychotic disorders usually have relatively intact ACE-III scores, especially in episodic memory and visuospatial domains. When deficits appear, they are often mild, nonspecific, or variable across domains. These may be due to low engagement, changes in attention, or symptom burden, rather than progressive disease [38]. A lack of stable, syndrome-specific patterns on ACE-III suggests against primary neurodegeneration, particularly early on.

ACE-III is especially helpful for diagnosing patients with early symptom onset. Some neurodegenerative conditions, such as bvFTD, start in mid-adulthood with marked behavioral changes, disinhibition, or paranoid ideas. These can resemble late-onset schizophrenia or other primary psychotic disorders [4]. ACE-III may reveal early, strong impairment in executive functions (like the serial sevens task or clock drawing) and verbal fluency, while memory and visuospatial skills remain relatively intact. This cognitive pattern points to frontotemporal neurodegeneration, even if psychiatric features dominate [77].

Importantly, ACE-III should not be interpreted in isolation but rather integrated with clinical history, symptom chronology, functional decline, and longitudinal observation. However, when applied systematically, ACE-III can serve as an early decision-support tool, prompting timely referral for further diagnosis when cognitive profiles are suggestive of neurodegeneration [8,10]. Conversely, relatively preserved multidomain performance may support a primary psychiatric diagnostic pathway while pointing to the need for ongoing cognitive monitoring. 

An additional diagnostic challenge arises in patients with mixed neuropathology, such as Alzheimer’s disease coexisting with vascular changes or Lewy body pathology. In such cases, ACE-III profiles may not conform to a single syndrome-specific pattern but instead present as overlapping or atypical cognitive profiles.

For example, a patient may demonstrate both memory impairment characteristic of Alzheimer’s disease and visuospatial deficits typical of Lewy body disease. In these situations, ACE-III findings should be interpreted cautiously and always integrated with neuroimaging, biomarkers, and longitudinal clinical data.

Biomarker-based diagnostic procedures include cerebrospinal fluid analysis of amyloid-β and tau proteins, amyloid and tau positron emission tomography (PET), and structural neuroimaging such as magnetic resonance imaging (MRI), assessing hippocampal atrophy and vascular changes. These biomarkers provide critical support for etiological diagnosis and should be considered when cognitive profiles are ambiguous.

In this context, the ACE-III serves as more than a screening instrument; it acts as a clinically informative bridge between psychiatry and neurology [18]. Its capacity to objectify cognitive patterns in patients presenting with psychotic symptoms enhances diagnostic confidence, reduces the risk of misclassification, and supports more appropriate, etiology-driven management strategies across the adult lifespan.

### 7.4. ACE-III in Differentiating Mild Cognitive Impairment from Primary Psychotic Disorders

Distinguishing MCI from primary psychotic disorders represents a significantly complex diagnostic scenario, as both conditions may present with subtle cognitive changes, preserved functional independence, and prominent psychiatric symptoms. Psychotic symptoms occurring in individuals without apparent dementia may raise concern for prodromal neurodegenerative disease; however, they may also reflect primary psychotic disorders, affective psychosis, or stress-related cognitive inefficiency. Accurate differentiation is essential, as MCI carries a substantially increased risk of progression to dementia, whereas primary psychotic disorders follow a different clinical course and management pathway.

MCI is defined by objective cognitive impairment greater than expected for age and education, with relative preservation of activities of daily living and absence of dementia. Importantly, neuropsychiatric symptoms—including depression, anxiety, apathy, and psychotic symptoms—are common in MCI and may precede or accompany cognitive decline [55]. In contrast, primary psychotic disorders are characterized by psychotic symptoms as the core clinical feature, while cognitive deficits—although frequently present—are typically non-progressive, less characteristic, and do not reflect a neurodegenerative process [78].

In this context, multidomain cognitive assessment using the ACE-III provides clinically relevant information that goes beyond global screening. In MCI individuals, ACE-III commonly reveals selective impairment in one or more cognitive domains—most frequently attention and episodic memory, or visuospatial abilities—while overall performance remains above dementia cut-off thresholds. Crucially, these deficits tend to be internally consistent and reproducible, in accordance with known prodromal patterns of neurodegenerative disease, particularly AD [18,26,31].

By contrast, patients with primary psychotic disorders typically demonstrate relatively preserved performance across ACE-III domains or show mild, inconsistent deficits that do not conform to a recognizable neurodegenerative profile. When present, cognitive difficulties in psychosis are commonly impacted by attentional fluctuations, reduced test engagement, or active psychotic symptoms rather than structural brain pathology. Episodic memory and visuospatial functioning—domains particularly sensitive to early neurodegeneration—are frequently intact in primary psychotic disorders, providing an important point of differentiation from MCI [78].

Longitudinal assessment further strengthens the diagnostic utility of ACE-III. In MCI, repeat testing often demonstrates a gradual decline within affected domains or progression toward a more global impairment pattern, supporting a neurodegenerative trajectory. In contrast, cognitive performance in primary psychotic disorders tends to remain relatively stable over time, even in the presence of persistent psychotic symptoms [79]. Thus, ACE-III may serve not only as a cross-sectional diagnostic tool but also as a tool for monitoring cognitive change over time.

Importantly, psychotic symptoms in MCI do not necessarily indicate imminent conversion to dementia and should be interpreted within the more extensive cognitive and functional context. However, the presence of specific pattern of impairment on ACE-III—particularly in memory or visuospatial processing—should prompt careful etiological evaluation and longitudinal follow-up. Conversely, the absence of objective cognitive impairment on ACE-III in individuals presenting with psychosis supports consideration of a primary psychiatric diagnosis while pointing to the need for ongoing cognitive surveillance.

Overall, ACE-III supports the differentiation between MCI and primary psychotic disorders by identifying selective, syndrome-consistent cognitive deficits characteristic of prodromal neurodegenerative disease. When combined with clinical history, functional assessment, and longitudinal observation, ACE-III strengthens diagnostic accuracy in patients presenting with psychotic symptoms without established dementia.

### 7.5. M-ACE in the Differentiation Between Primary Psychosis and Neurodegeneration

Differentiating primary psychotic disorders from early neurodegenerative disease is particularly challenging in late-life presentations, where psychotic symptoms might predominate, and cognitive impairment may be subtle or fluctuating. In this context, the M-ACE serves as a useful initial screening tool, supporting early etiological reasoning and guiding further diagnostic evaluation.

M-ACE provides a rapid assessment of global cognition while sampling domains commonly affected early in neurodegeneration, including episodic memory, verbal fluency, attention, and visuospatial abilities [42]. In individuals with emerging neurodegenerative disease, M-ACE performance is often objectively reduced even at prodromal stages, whereas patients with primary psychotic disorders typically show relatively preserved scores, with any deficits tending to be mild, inconsistent, or state-dependent [80].

From a differential diagnostic perspective, an abnormal M-ACE result in a patient presenting with psychosis should prompt consideration of a neurodegenerative etiology and referral for comprehensive cognitive profiling using ACE-III and standard neuropsychological testing. Conversely, a normal or near-normal M-ACE score—particularly in the absence of functional decline—supports, though does not confirm, a primary psychiatric diagnosis and stresses the value of longitudinal monitoring.

Importantly, M-ACE may detect domain vulnerabilities atypical for primary psychosis but characteristic of neurodegeneration, such as visuospatial or fluency impairment suggestive of Lewy body or frontotemporal pathology, or impaired recall consistent with prodromal AD [26,37,42]. As a triage instrument within a stepped diagnostic pathway, M-ACE helps identify patients requiring urgent neurocognitive evaluation and reduces the risk of diagnostic delay and misclassification.

## 8. Discussion

### 8.1. Clinical Implications of ACE-III in Dementias

Although ACE-III demonstrates strong clinical utility, its limitations must be recognized. The available evidence is heterogeneous, with many studies involving small samples and variable methodologies, limiting comparability. Furthermore, the capacity of ACE-III to clearly differentiate between dementia subtypes is compromised in advanced stages or mixed pathology, reducing its specificity.

Conflicting findings mean results must be interpreted with caution. Clinicians should integrate cognitive assessment with clinical, functional, and biomarker data.

Multidomain cognitive assessment plays a central role in the diagnostic evaluation of patients presenting with psychotic symptoms in later life. In this context, the ACE-III and its brief derivative, the M-ACE, offer complementary benefits that extend beyond conventional cognitive screening and meaningfully support etiological differentiation.

The ACE-III helps distinguish dementia-related psychosis from late-onset schizophrenia spectrum and mood disorders with psychotic features. Though psychotic symptoms may overlap [4,66], dementia-related psychosis usually comes with specific cognitive impairments tied to neurodegeneration [37]. ACE-III examines memory, attention, verbal fluency, language, and visuospatial abilities. This allows clinicians to identify cognitive profiles associated with AD, DLB, FTD, or VaD. In contrast, primary psychotic and mood disorders often show mostly preserved cognition or mild, inconsistent deficits that do not progress by domain [78].

Recognizing these profiles guides both diagnosis and treatment. It helps decide whether to use antipsychotic medication in DLB, predict disease course, and counsel patients and caregivers.

Repeated ACE-III testing builds diagnostic confidence by tracking cognitive decline over time. Progressive, domain-specific decline supports neurodegeneration. Stable cognition despite ongoing psychosis suggests a primary psychiatric disorder. Longitudinal assessment is especially valuable in early or atypical cases, where a single evaluation may not tell the full story. Serial ACE-III testing offers an objective way to monitor disease progression and refine the diagnosis as new information emerges.

It should also be noted that ACE-III’s ability to differentiate between dementia subtypes is inconsistent, especially in advanced disease stages when cognitive impairment becomes global. Overlapping cognitive patterns may occur in mixed or atypical cases, further limiting the diagnostic specificity of the ACE-III. These factors underscore the need for cautious interpretation and integration of ACE-III results with clinical, functional, and biomarker data.

Selected longitudinal studies relevant to psychosis-associated neurodegeneration and ACE-based cognitive assessment are summarized in Table 3. Collectively, these findings support the importance of repeated cognitive evaluation and demonstrate that longitudinal cognitive trajectories may provide diagnostically meaningful information beyond single-time-point assessment.

Overall, available longitudinal evidence suggests that progressive and syndrome-consistent cognitive decline provides stronger support for neurodegenerative pathology than isolated cross-sectional cognitive findings.

### 8.2. Future Perspectives

Future research should employ larger, well-defined cohorts and longitudinal designs to evaluate ACE-III profiles in psychosis-related neurodegeneration. Studies should move beyond single-test cut-offs and adopt profile-based, continuous diagnostic models. Larger investigations are required to assess specific and percentage-based ACE-III profiles in both psychiatric and neurological populations, particularly among individuals with late-life psychosis, atypical dementia syndromes, or genetic disorders. Integrating cognitive profiles with biomarkers and neuroimaging is likely to enhance diagnostic accuracy.

Further research should investigate the implementation of stepwise diagnostic pathways in clinical settings. Initiating assessment with M-ACE for triage, followed by ACE-III and targeted neuropsychological tests, may expedite diagnosis, improve referral accuracy, and reduce inappropriate antipsychotic prescribing. Studies should examine the impact of these diagnostic steps on clinical outcomes, treatment strategies, and caregiver burden.

Emerging digital tools and analytical techniques have the potential to enhance the clinical utility of the ACE-III. Automated scoring systems and visual representations of domain results, combined with machine learning applied to domain-level data, may support clinical decision-making. Explainable AI approaches could support interpretation of ACE-III and M-ACE by identifying transparent domain-level patterns, for example memory-led profiles suggestive of AD, visuospatial–attention profiles suggestive of DLB/PDD, or fluency–executive profiles suggestive of FTD. Importantly, such models should provide clinically interpretable explanations rather than black-box classifications. However, these methods must remain transparent, user-friendly, and subject to rigorous validation.

Future investigations should also address clinician education and interdisciplinary collaboration among psychiatry, neurology, and neuropsychology. Enhancing awareness of dementia-related psychosis and the capabilities and limitations of screening instruments may reduce misdiagnosis and improve patient care.

Such approaches, however, require external validation, methodological transparency, and integration with standard clinical assessment rather than replacement of clinical judgment.

A significant limitation in the existing literature is the limited number of longitudinal studies investigating the progression of ACE-III and M-ACE profiles in psychosis-related neurodegeneration. Future research should prioritize prospective longitudinal designs to elucidate how characteristic cognitive patterns evolve over time and predict conversion from mild cognitive impairment or late-life psychosis to dementia.

Serial ACE-III assessments may provide a sensitive means of detecting subtle cognitive decline and differentiating progressive neurodegenerative processes from relatively stable cognitive deficits characteristic of primary psychiatric disorders.

### 8.3. Limitations 

Several limitations must be considered when interpreting the clinical implications presented in this narrative review. First, ACE-III and M-ACE are screening instruments and do not substitute for comprehensive neuropsychological assessment or biomarker-based diagnostic procedures. Their specificity is limited, particularly in advanced disease stages, and they should primarily support etiological reasoning and risk stratification rather than serve as definitive diagnostic tools.

The specificity of ACE-III profiles diminishes as disease severity increases. In moderate to advanced dementia, cognitive impairment becomes global, restricting the ability to differentiate between dementia subtypes using domain-level patterns. In these instances, the ACE-III is most useful for staging and documenting overall cognitive decline.

In contrast, very early neurodegenerative diseases such as FTD may present with significant behavioral or psychotic symptoms despite normal ACE-III or M-ACE scores. Exclusive reliance on these scores can be misleading. Comprehensive longitudinal assessment, including clinical history, informant reports, and neuroimaging, is essential. Performance on these instruments is also affected by state-dependent factors such as active psychosis, mood symptoms, medication effects, reduced motivation, or sensory impairment. These influences are particularly pertinent in psychiatric populations and necessitate careful clinical judgment.

Other limitations include ceiling effects, particularly among highly educated individuals or those in the earliest stages of decline, where scores may appear normal despite underlying deficits [25]. Demographic factors such as age, education, and premorbid ability can also bias results, resulting in over- or underestimation of impairment. The current literature is further constrained by small sample sizes, variability in diagnostic criteria and cut-off values, and heterogeneity in study designs, all of which limit the direct comparability of findings. Additionally, few studies have specifically addressed psychosis-related populations, and longitudinal data remain scarce.

These constraints are partly attributable to the relatively recent introduction of the ACE-III. Although now widely adopted, it has been available for a shorter period than earlier cognitive assessment tools, limiting the scope of long-term research. Its application in non-English-speaking populations requires independent validation and cultural adaptation, which further slows the accumulation of comparable data. The synthesis of evidence is also affected by heterogeneity in study design, diagnostic approaches, and outcome measures, as well as by potential publication bias. The lack of meta-analytic methods restricts quantitative comparisons of diagnostic accuracy across instruments.

Despite these limitations, the available evidence supports the clinical utility of the ACE-III as an accessible multidomain instrument that enhances diagnostic reasoning, particularly when used longitudinally and in combination with complementary clinical data.

## 9. Conclusions

Accurate cognitive assessment is essential for the early detection and differential diagnosis of neurodegenerative disorders, particularly when psychotic symptoms such as delusions or hallucinations are present. Domain-specific scores from the ACE-III allow clinicians to identify overall cognitive impairment and to characterize cognitive strengths and weaknesses associated with different dementia syndromes.

Beyond its application in neurodegenerative disorders, the ACE-III may also aid in the assessment of individuals with primary psychotic disorders. Classic psychoses such as schizophrenia or schizoaffective disorder can manifest cognitive impairments; however, these deficits are typically non-progressive and less characteristic than those observed in dementias. In such cases, the ACE-III profile—characterized by relatively preserved memory and visuospatial function alongside mild attentional or fluency deficits—can assist in distinguishing these conditions from progressive neurodegenerative profiles. Although systematic studies of ACE-III in primary psychosis are limited, its comprehensive structure can reveal patterns that single global scores such as the MMSE or MoCA may not detect, particularly when assessments are repeated over time to differentiate static from progressive impairment. ACE-III scores alone are insufficient for subtype differentiation; without analysis of domain subscores, important distinctions, such as visuospatial versus memory or fluency deficits, may be missed. Therefore, profile analysis and cross-domain comparison are essential to maximize the diagnostic utility of ACE-III in both neurodegenerative disorders with psychosis and in efforts to exclude primary psychiatric conditions.

The ACE-III should be considered a supportive instrument for cognitive profiling rather than a standalone diagnostic tool. Its results must be interpreted within a comprehensive clinical and biomarker-based context.

## Figures and Tables

**Table 1:** Comparative cognitive and psychotic features across neurodegenerative disorders relevant to ACE-III interpretation.

Disorder/ Variant	Dominant Cognitive Profile	Typical Psychotic Features	Characteristic Behavioral/ Neuropsychiatric Features	Distinguishing Clinical Features Relevant to ACE-III Interpretation
Typical Alzheimer’s disease (AD)	Episodic memory impairment with later multidomain involvement	Delusions more common than hallucinations; persecutory beliefs, misidentification syndromes	Apathy, depression, anxiety, progressive functional decline	Memory-led impairment with relatively preserved visuospatial and executive functions early
MCI/Mild Behavioral Impairment (MBI)	Subtle, selective deficits (often memory or attention) with preserved daily functioning	Mild or intermittent delusions/hallucinations; psychosis uncommon but clinically significant	Anxiety, depression, apathy, irritability, emerging behavioral changes	Domain-specific deficits may precede dementia; longitudinal decline more informative than single assessment
Posterior cortical atrophy (PCA)	Visuospatial and perceptual dysfunction	Early visual hallucinations possible	Visual disorientation, reading difficulty, perceptual disturbances	Severe visuospatial impairment with relatively preserved memory and language early
Logopenic variant PPA (lvPPA)	Language and verbal working memory impairment	Psychosis uncommon	Word-finding pauses, phonological errors	Language-led impairment with relatively preserved visuospatial abilities
Frontal variant AD (fvAD)	Executive dysfunction, fluency, attention, and memory impairment	Delusions, behavioral disinhibition, psychiatric-like presentation	Executive dysfunction, apathy, irritability, socially inappropriate behavior	Combined executive and memory impairment may mimic bvFTD but with stronger episodic memory involvement
AD-related corticobasal syndrome (AD-CBS)	Executive, attentional, visuospatial, and praxis dysfunction	Psychosis uncommon but may occur in advanced disease	Apraxia, asymmetric motor symptoms, attentional dysfunction	Mixed profile with early memory impairment and visuospatial dysfunction may suggest underlying AD pathology
Behavioral variant FTD (bvFTD)	Executive dysfunction and impaired fluency	Suspiciousness, somatic delusions, impaired insight	Disinhibition, apathy, compulsive behavior, emotional blunting	Executive–fluency impairment disproportionate to memory deficits
Semantic variant PPA (svPPA)	Semantic memory and naming impairment	Rare psychosis; rigid or bizarre beliefs	Loss of word meaning, altered food preference, compulsive behaviors	Severe semantic deficits with relatively preserved visuospatial abilities
Nonfluent/agrammatic PPA (nfvPPA)	Nonfluent speech, agrammatism, speech apraxia	Psychosis uncommon	Effortful speech, apraxia of speech	Language production deficits dominate profile
C9orf72-associated FTD	Executive and behavioral dysfunction	Prominent hallucinations and delusions; schizophrenia-like presentations	Bizarre behavior, impaired insight, apathy	Psychosis may precede overt dementia despite mild cognitive deficits
Dementia with Lewy bodies (DLB)	Visuospatial and attentional impairment with fluctuating cognition	Early recurrent visual hallucinations, Capgras syndrome, illusions	REM sleep behavior disorder, fluctuations, Parkinsonism	Early visuospatial deficits and hallucinations strongly support DLB
Parkinson’s disease dementia (PDD)	Executive and attentional dysfunction with reduced fluency	Visual hallucinations, passage hallucinations, paranoid delusions	Psychomotor slowing, sleep disturbances	Executive dysfunction more prominent than episodic memory impairment
Vascular Dementia (VaD)	Heterogeneous “patchy” executive–attentional profile	Variable delusions and hallucinations; fluctuating symptoms	Psychomotor slowing, gait disturbance, affective symptoms	Uneven cognitive profile and stepwise decline suggest vascular pathology

**Table 2:** Comparison of ACE-III with commonly used cognitive, functional, and neuropsychiatric assessment tools in neurodegenerative disorders with psychotic symptoms.

Instrument	Primary Assessment Domain	Main Strengths	Main Limitations	Clinical Role in Differential Diagnosis
ACE-III [18]	Multidomain cognition (attention, memory, fluency, language, visuospatial abilities)	Provides detailed cognitive profiling; sensitive to atypical and non-amnestic presentations; supports syndrome-oriented interpretation	Longer administration time than MMSE/Mini-ACE; performance influenced by education and language; less specific in advanced dementia	Core cognitive profiling tool supporting etiological differentiation between dementia syndromes and primary psychotic disorders
M-ACE [42]	Brief multidomain cognitive screening	Rapid administration; suitable for initial screening and busy clinical settings; sensitive to early cognitive impairment	Less detailed domain analysis; reduced ability to characterize syndrome-specific profiles	First-line cognitive screening and triage tool indicating need for comprehensive assessment
MMSE [12]	Global cognitive screening	Widely used; rapid and simple administration; useful for general cognitive estimation and longitudinal staging	Limited sensitivity to executive and visuospatial dysfunction; ceiling effects; poor differentiation of atypical dementias	General cognitive screening and severity estimation
MoCA [13]	Global cognition with emphasis on executive function	Greater sensitivity to mild cognitive impairment and executive dysfunction than MMSE	Less detailed qualitative profiling than ACE-III; lower specificity in some clinical populations	Screening for mild cognitive impairment and early cognitive decline
CDR [17]	Dementia severity and functional staging	Standardized staging of dementia severity; useful for monitoring disease progression	Does not provide detailed cognitive domain analysis	Functional staging and assessment of dementia severity
FAQ [16]	Instrumental activities of daily living	Sensitive to functional decline associated with dementia; useful in distinguishing MCI from dementia	Does not assess cognition directly; influenced by physical disability and caregiver report	Functional assessment supporting diagnostic classification
NPI-Q [16]	Behavioral and neuropsychiatric symptoms	Assesses hallucinations, delusions, agitation, mood, and caregiver burden; clinically relevant in psychosis-associated dementias	No direct cognitive assessment; symptom severity may fluctuate	Characterization of neuropsychiatric and psychotic symptoms
Comprehensive neuropsychological assessment	Detailed multidomain cognition	High diagnostic precision; extensive assessment of cognitive strengths and weaknesses	Time-consuming; requires specialist administration; less feasible in routine screening	Gold-standard cognitive characterization and complex differential diagnosis

**Table 3:** Selected longitudinal studies relevant to ACE-based assessment in psychosis-related neurodegeneration.

Study	Population	Design/ Follow-Up	Main Findings	Relevance to ACE-III and Differential Diagnosis
Kaczmarek et al., 2026 [81]	Older adults with normal cognition, MCI, and dementia	13-month longitudinal follow-up	Significant transitions between diagnostic categories (normal cognition → MCI → dementia); ACE-III and Mini-ACE sensitive to cognitive progression	Supports utility of ACE-based tools in monitoring progression and early cognitive change
Carrick et al., 2025 [37]	Patients with various neurodegenerative dementias	Repeated ACE-III assessments (annual follow-up)	Progressive decline in ACE-III scores (~7–9 points/year); changes ≥5 points considered clinically meaningful	Demonstrates longitudinal sensitivity of ACE-III to progressive cognitive deterioration
Zhang et al., 2025 [82]	Patients with AD, bvFTD, and semantic dementia	Longitudinal follow-up (~2.4 years)	Distinct trajectories of decline across ACE-III cognitive domains depending on syndrome	Highlights value of domain-specific longitudinal cognitive profiling for differential diagnosis
Foxe et al., 2022 [83]	Primary progressive aphasia variants	Longitudinal follow-up (~6 years)	Distinct trajectories of decline across PPA variants; fastest progression in logopenic variant	Demonstrates heterogeneity of longitudinal ACE-III profiles across neurodegenerative syndromes
Wearn et al., 2020 [84]	Cognitively healthy older adults	12-month follow-up	Subtle memory impairment predicted subsequent decline on ACE-III	Suggests ACE-III sensitivity to preclinical neurodegenerative change
Schubert et al., 2016 (ACE-R study) [80]	Patients with bvFTD and AD	Longitudinal cognitive assessment	Faster cognitive decline observed in bvFTD compared with AD	Supports the role of progression rate and longitudinal assessment in distinguishing dementia syndromes
Rittman et al., 2013 (ACE-R study) [77]	Parkinsonian syndromes (PD, PSP, CBD)	Longitudinal follow-up (~18 months)	Greatest cognitive decline observed in corticobasal degeneration; fluency measures highly discriminative	Earlier Addenbrooke’s version supporting sensitivity of domain-level cognitive assessment to progression and syndrome differentiation

## Data Availability

No new data were created or analyzed in this study. Data sharing is not applicable to this article.

## Abbreviations

ACE-III	Addenbrooke’s Cognitive Examination III
AD	Alzheimer’s disease
APP	Amyloid protein precursor
BPSDs	behavioural and psychological symptoms of dementia
BvFTD	behavioural variant frontotemporal dementia
C9orf72	chromosome 9 open reading frame 72
CDR	Clinical Dementia Rating
DLB	Dementia with Lewy Bodies
FAQ	Functional Activities Questionnaire
FTD	Frontotemporal Dementia
FTLD	Frontotemporal Lobar Degeneration
M-ACE	Mini-Addenbrooke’s Cognitive Examination
MBI	Mild Behavioral Impairment
MCI	Mild Cognitive Impairment
MMSE	Mini-Mental State Examination
MoCA	Montreal Cognitive Assessment
MRI	Magnetic Resonance Imaging
NPI-Q	Neuropsychiatric Inventory Questionnaire
PANSS	Positive And Negative Syndrome Scale
PD	Parkinson’s Disease
PDD	Parkinson’s Disease Dementia
PET	Positron Emission Tomography
PPA	Primary Progressive Aphasia
PSEN	Presenilin
REM	Rapid Eye Movement
VaD	Vascular Dementia
